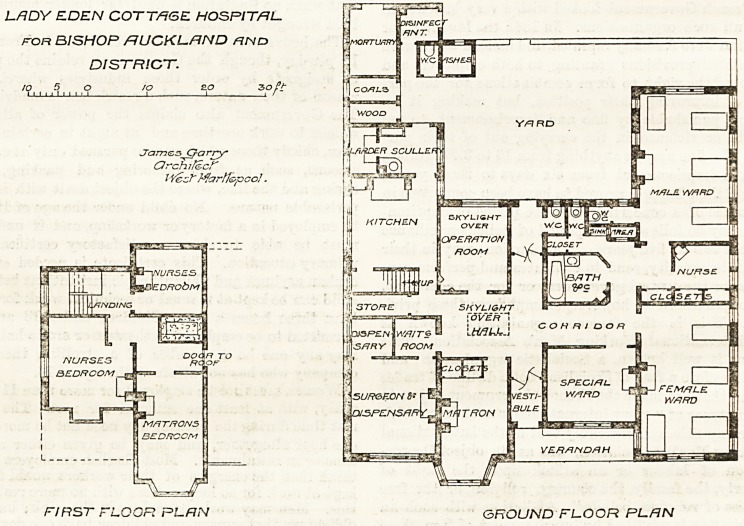# Hospital Construction

**Published:** 1900-01-27

**Authors:** 


### The Institutional Workshop.

### HOSPITAL CONSTRUCTION.

### THE BISHOP AUCKLAND COTTAGE HOSPITAL.

The advisableness of erecting a cottage hospital in Bishop Auckland for the treatment of accidents happening in the neighbouring mines was for a long time talked of, and the subject seems to have been opened by the late Dr. Thwaites, but not much was done during his lifetime. Subsequently Dr. Wardle collected some money which was placed to a hospital fund in the bank. Enthusiasm at that time was not great, and again the project fell to the ground. It was lately revived and carried to a successful issue by Lady Eden; and it must be satisfactory for everyone to reflect that her work has been recognised in the most suitable way by naming the institution "The Lady Eden Hospital." The coal companies in the neighbourhood subscribed, as did also the private inhabitants, and altogether a fund exceeding £2,000 was obtained. In addition to subscriptions there were many gifts of kind. For instance, Mr. Everitt supplied the bedsteads. The funds have been very carefully managed, and, in place of the usual debt, the hospital began its career of usefulness with a balance of £338 in its favour, and it has been assured support from the workmen of the collieries.

The hospital has been erected opposite the workhouse at Cockton Hill, on a site consisting of 1,500 square yards, which has been valued at £500, but which was obtained at a much lower sum from the Ecclesiastical Commissioners. The hospital is placed about 30 yards from the high road. The principal entrance faces west, and is opposite the workhouse. The building was formally opened by Lord Rosebery in September, 1899. On the right of the entrance, and also facing west, is the special ward. It has only one window, and this looks into a verandah. Facing south are the two main wards, each containing three beds. These dormitories are placed in line with a nurses' room between them, so that supervision is rendered easy. The men's dormitory is well-arranged, having abundance of light and efficient cross-ventilation; but the women's ward is not so well-provided for in these respects, and manifestly the west end of the ward ought to have been drawn out 10 ft. beyond the present west elevation. The sanitary arrangements are incorporated with the main building, instead of being placed in the

FIRST FLOOR PLAN

GROUND FLOOR PLAN

yard, or as spurs from the dormitories and connected by efficient cross-ventilated passages. It does not seems clear how the bath-room is either lighted or ventilated. If by a skylight only, it is hardly likely to be successful. The operation room is of fair size, has a large window facing east, and very properly, a large roof-light in addition. The room is placed in close proximity to the kitchen, and the scullery window is at right angles; but then everyone knows that in a small hospital it is by no means easy to place everything just where one would like it to be.

The matron's room, surgeon's room, kitchen, mortuary, and disinfecting room are all well enough arranged. The first floor contains sleeping accommodation for the resident staff.

The building is in the so-called domestic style of architecture, and the general effect of the west elevation is pleasing. The warming is by open fireplaces, Portland cement has been used in ceilings, walls, and corridors.

The chief points to which we would take exception in this plan are the central position of the sanitary arrangements and the opening of all the wards, the nurses' room, the bath-room, &c., into one corridor in such a manner that there is likely to be considerable intermingling of air between these different rooms unless some means of ventilation exists which is not shown in the drawing.

### THE LEICESTER SQUABBLE.

Except for the actual payment of the costs by the defendants the Leicester vaccination squabble has come to an end. Mr. Lawson Walton, Q.C., applied to the Court last Monday that the writ of attachment against 22 members of the Leicester Board of Guardians should be discharged; and he laid before the Court affidavits showing not only the desire of the defendants not to be disrespectful to the Court, but that they had taken practical steps to comply with its directions. The judge, however, was not satisfied until he had received affidavits from the Guardians individually, and after hearing these he said, "I understand that the effect of these affidavits is that all the defendants have submitted, and that none of them can now say, 'I stood out against the Court to the very last.'" To this Mr. Lawson Walton, on behalf of the defendants, answered, "I submit that that is the case." Mr. Justice Channell, in delivering judgment, said that "if the Local Government Board had pressed the Court to inflict a further punishment than the payment of the costs of the proceedings they might have been inclined to do so. But as they had not taken this course, and the question of the dignity of the Court was not involved, but only a question of doing the thing required to be done, the writs would be discharged upon the payment by the defendants, individually, of the costs of the proceedings, including the costs of the mandamus. In the ordinary way the last-mentioned costs would fall upon the ratepayers; but, inasmuch as the subsequent conduct of the defendants showed that the course they took in those proceedings was not adopted as part of their public duties, the Court thought that they ought to pay personally the costs incurred in those proceedings. The costs would be taxed as between solicitor and client." Mr. Justice Bucknill concurred. Thus ends a most discreditable attempt to use a public position for the furtherance of private views. In this particular case the object of attack was vaccination, and there are a sufficient number of ignorant people in the world to have enabled those who defied the law to obtain some sort of a following. But vaccination is not the only question on which there are differences of opinion, and it must be remembered that, if it were once allowed that persons could accept office on public boards and then refuse to carry out the statutory duties attaching to such office, the whole machinery of local government might be brought to a standstill. It is well that the law has put its foot down firmly on such attempts.

## Figures and Tables

**Figure f1:**